# Relationship Between Anemia, and Serum Testosterone Levels in Chronic Obstructive Pulmonary Disease Patients: A Cross-sectional Study -

**DOI:** 10.31661/gmj.v14i.4088

**Published:** 2025-11-17

**Authors:** Alireza Rezvani, Eshagh Javidan, Shakiba Karami, Roohollah Rahbani

**Affiliations:** ^1^ Hematology Research Centre, Shiraz University of Medical Sciences, Shiraz, Iran; ^2^ Rajaie Cardiovascular Research Center, Iran University of Medical Sciences, Tehran, Iran; ^3^ I.M. Sechenov First Moscow State Medical University, Moscow, Russia

**Keywords:** Chronic Obstructive Pulmonary Disease (COPD), Anemia, Serum Testosterone Levels, Hormonal Imbalances, Comorbidities

## Abstract

**Background:**

Chronic obstructive pulmonary disease (COPD) is a complex condition that can be associated with various comorbidities, including anemia and hormonal imbalances. The relationship between COPD, anemia, and serum testosterone levels has not been thoroughly investigated. To examine the relationship between COPD, anemia, and serum testosterone levels in patients with COPD.

**Materials and Methods:**

This cross-sectional study was conducted at Shahid Faghihi and Namazi hospitals in Shiraz, Iran, and included 43 patients with COPD who were admitted to internal medicine and emergency departments between autumn 2018 and autumn 2019. Patients were evaluated for anemia, and serum testosterone levels were measured. Data were analyzed using SPSS version 22.

**Results:**

The study included 27 males (62.8%) and 16 females (37.2%). The prevalence of anemia was 48.1% (13/27) in males and 50% (8/16) in females. Total testosterone was significantly higher in males (M=1.74, SD=1.85) compared to females (M=0.47, SD=0.69), P.001. The study found that females with anemia had significantly higher mean testosterone total levels compared to those without anemia (p=0.047). However, no significant differences were found in testosterone levels between males with and without anemia. While females with anemia had a mean total testosterone level of 0.32 (SD=0.27), while those without anemia had a mean level of 0.61 (SD=0.91), with a significant P-value of 0.047.

**Conclusion:**

This study suggests a potential relationship between anemia and serum testosterone levels in female patients with COPD. Further research is needed to confirm these findings and explore the underlying mechanisms.

## Introduction

Chronic obstructive pulmonary disease (COPD) is one of the common causes of morbidity, reduced quality of life, and mortality in adults worldwide, including Iran, and imposes a significant financial burden on the healthcare system and patients [1][2]. According to available data, due to reasons such as increased exposure to environmental pollution, mortality from COPD has increased over the past 15 years in Iran [3]. COPD is one of the main causes of mortality in both developed and developing countries, and its mortality rate is increasing [4]. This disease is the fourth leading cause of death in the United States, after cardiovascular disease, cancer, and cerebrovascular diseases, and GOLD estimates suggest that by 2020, it will become the third leading cause of death worldwide, rising from sixth place [5].

Individuals with COPD often suffer from comorbidities, which reduce their quality of life and affect their prognosis [6][7]. Notably, these comorbidities are often undiagnosed or not properly treated [6]. One of the concurrent disorders in people with chronic obstructive pulmonary disease (COPD) is anemia. According to available data, up to 33% of people with COPD also suffer from anemia [8]. It seems that anemia of chronic disease is the most common type of anemia in this group of patients [8]. One of the problems faced by individuals with COPD is hormonal disorders. For example, it has been shown that a significant percentage of men with COPD have hypogonadism and low serum testosterone levels [9]. This can lead to other complications such as sexual dysfunction, loss of muscle mass and strength (including respiratory muscles), and loss of bone mineral density and osteoporosis, ultimately affecting their prognosis [9][10][11].

Androgens play a crucial role in the development and maintenance of male reproductive and sexual functions, body composition, bone health, and behavior. A decrease in serum testosterone levels is referred to as hypogonadism [12] and can contribute to muscle wasting in patients with COPD. In various studies, decreased testosterone levels have been reported in patients with COPD, and in some cases, it has been associated with the severity of symptoms and the degree of airflow limitation, as measured by forced expiratory volume in one second (FEV1) [13][14][15]. However, in other studies, no such association has been found [16]. According to a review study by Boutou et al., up to 38% of patients with COPD suffer from anemia, and one of the factors considered as a cause of anemia in this study was the decrease in serum testosterone levels in these patients [17]. However, as a review of existing literature shows, the relationship between the COPD, anemia, and serum testosterone levels in patients with COPD has not been precisely evaluated. Considering this and the importance of identifying and treating underlying factors in this group of patients, we aimed to address this issue in this study.

## Materials and Methods

This cross-sectional study included all patients diagnosed with COPD who were admitted to the internal medicine and emergency departments of Shahid Faghihi and Namazi hospitals in Shiraz from autumn 2018 to autumn 2019, and patients received standard treatment during the study period. Clinical diagnosis of COPD was confirmed after patients being evaluated by a multidisciplinary team of pulmonologists, radiologists, and internal medicine specialists. The study population consisted of patients with COPD who were admitted to the internal medicine and emergency departments of the aforementioned hospitals. Only patients between the ages of 18 and 70 years were included in the study. Patients with asthma and other pulmonary diseases unrelated to COPD were excluded from the study. Various blood parameters, including complete blood count (CBC) and serum testosterone levels, were measured. Hemoglobin levels below 13 g/dl for men and below 12 g/dl for women were considered anemic.

All study participants underwent measurement of total serum testosterone levels and complete blood cell count using an Automated H Analyzer with informed consent. Laboratory evaluations were performed at university-approved centers. COPD diagnosis was based on American Thoracic Society criteria, including forced expiratory volume in 1 second (FEV1) <80% predicted, FEV1/forced vital capacity (FVC) ratio <0.7, and a change in FEV1 of <200 mL or <12% after bronchodilator testing. Clinical diagnosis was also taken into account.

Data were analyzed using IBM SPSS Statistics for Windows, version 22 (IBM Corp., Armonk, N.Y., USA). In the inferential section, the normality of the distribution of variables was first assessed. If the normality assumption was met, parametric methods were used; otherwise, non-parametric methods were employed. The Kolmogorov-Smirnov test was used to assess the normality of the distribution of variables. Descriptive statistics, including mean, standard deviation, minimum, and maximum (for quantitative data), frequency, percentage, and cumulative percentage (for qualitative data), were used to analyze the data. Non-parametric tests, including the Mann-Whitney U test, were used for statistical analysis. Mean serum testosterone levels were compared between groups, regardless of age and disease severity, and within each age and severity group.

## Results

**Table:** Table2. Prevalence of Anemia by Gender

**Gender**	**Anemic *n* (%) **	**Non-Anemic *n* (%) **	**Total *n* (%) **
Male	13 (48.1%)	14 (51.9%)	27 (62.8%)
Female	8 (50.0%)	8 (50.0%)	16 (37.2%)
**Total**	21 (48.8%)	22 (51.2%)	43 (100%)

**Table:** Table1. Comparison of Testosterone Levels Between Genders

**Testosterone Type**	**Gender**	***n***	**Mean**	**SD**	**P-value**
**Free**	**Male**	27	4.15	3.06	0.91
	**Female**	16	4.39	8.78	
**Total**	**Male**	27	1.74	1.85	<.001
	**Female**	16	0.47	0.69	

**Table:** Table3. Mann-whitney Test of Testosterone levels by Gender Based on the Anemia Status

**Gender**		**Anemia**	**n**	**Mean**	**SD**	**P-value**
	Testosterone Free	Yes	13	4.75	3.21	0.423
**Male**		No	14	3.59	2.91	
	Testosterone Total	Yes	13	2.25	2.00	0.327
		No	14	1.27	1.66	
	Testosterone Free	Yes	8	6.35	12.11	0.885
**Female**		No	8	2.42	1.45	
	Testosterone Total	Yes	8	0.32	0.27	0.047
		No	8	0.611	0.91	

**Figure-1:**
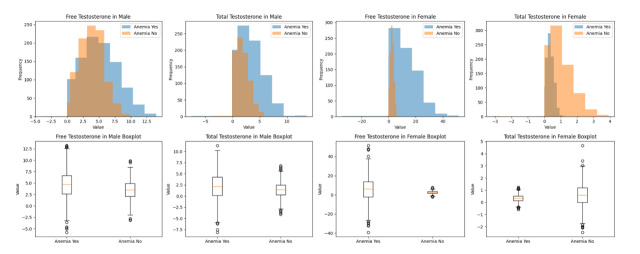
Figure 1. histogram and box plot of free and total testosterone of study participants

This study examined 43 patients with COPD (27 male; 16 female). Twenty-one (48.83%) were anemic. Mean age of participant was 63.17±5.7 years. An independent samples t-test revealed no significant difference in age between males (63.4±5.9 years) and females (62.8±5.4 years), t(41)=0.35, P=0.728. An independent samples t-test indicated no significant difference in age between anemic (64.2±6.1 years) and non-anemic participants (62.1 ± 5.3 years), t (41)=1.24, P=0.222.

A chi-square test of independence found no significant association between gender and anemia prevalence, χ² (1, N=43)=0.02, P=.887. Anemia rates were similar in males (48.1%) and females (50.0%).

A Mann-Whitney U test revealed no significant difference in free testosterone levels between males (M=4.15, SD=3.06) and females (M=4.39, SD=8.78), U=210, P=.910. However, total testosterone was significantly higher in males (M=1.74, SD=1.85) compared to females (M=0.47, SD=0.69), U=85, P<.001 (Table-1). For males (n=27), Figure-1 shows that in the group of male patients with anemia (n=13), the mean testosterone free and total levels were 4.75 and 2.25, respectively, with standard deviations of 3.21 and 2.91, and ranges of 1.10-10.40 and 0.10-8.50, respectively. The 25th, 50th, and 75th percentiles for testosterone free were 1.90, 3.60, and 7.35, respectively, and for testosterone total were 0.15, 0.50, and 1.95, respectively. In contrast, in the group of males without anemia (n=14), the mean testosterone free and total levels were 3.59 and 1.27, respectively, with standard deviations of 2.00 and 1.66, and ranges of 1.30-7.50 and 0.10-5.00, respectively.

The 25th, 50th, and 75th percentiles for testosterone free were 2.20, 3.20, and 6.80, respectively, and for testosterone total were 0.10, 0.60, and 3.00, respectively. For females, Figure-1 shows that in the group of females with anemia (n=8), the mean testosterone free and total levels were 6.35 and 0.32, respectively, with standard deviations of 12.11 and 0.27, and ranges of 0.70-36.20 and 0.10-0.80, respectively. The 25th, 50th, and 75th percentiles for testosterone free were 1.20, 2.00, and 2.90, respectively, and for testosterone total were 0.12, 0.35, and 0.70, respectively. In contrast, in the group of females without anemia (n=8), the mean testosterone free and total levels were 2.42 and 0.61, respectively, with standard deviations of 1.45 and 0.91, and ranges of 1.00-5.80 and 0.10-3.00, respectively. The 25th, 50th, and 75th percentiles for testosterone free were 1.25, 2.10, and 200, respectively, and for testosterone total were 0.10, 0.30, and 0.60, respectively. Based on the chi-square test, there was no significant difference in rate of anemia between males (48.14%) versus females (50%), P=0.991. Table-2 presents the frequency distribution of testosterone levels by gender based on anemia status, revealing notable differences between females with and without anemia. For males, the mean testosterone free level was 4.75 (SD=3.21) in the anemia group and 3.59 (SD=2.91) in the non-anemia group, with a non-significant P-value of 0.423 based on the Mann-Whitney test. Similarly, the mean testosterone total level was 2.25 (SD=2.00) in the anemia group and 1.27 (SD=1.66) in the non-anemia group, with a non-significant P-value of 0.327 (Table-3). In contrast, females with anemia had a significantly higher mean testosterone total level of 0.32 (SD=0.27) compared to those without anemia, who had a mean level of 0.61 (SD=0.91), with a significant P-value of 0.047. Additionally, females with anemia had a mean testosterone free level of 6.35 (SD=12.11), while those without anemia had a mean level of 2.42 (SD=1.45), with a non-significant P-value of 0.885.

## Discussion

The findings of this study showed that out of 43 people who were studied, 16 were female (37.21%) and 27 were male (62.79%), and there was no significant difference in the rate of anemia between males (48.14%) and females (50%), P=0.991. The higher prevalence of anemia in men in this study is similar to the findings of Halvani et al. [18]. The results are also similar to the studies conducted in 2006 in Yazd [19] and in 2006 in England [20], as well as reference books on respiratory diseases [21][22].

Anemia in patients with COPD can exacerbate the disabilities and limitations caused by the disease and have a negative impact on the quality of life of these patients. The findings of the Mann-Whitney test showed that there was a significant correlation only among women in the total testosterone test (P=0.047), but no significant difference was observed in other groups (P>0.05).

Hashemi Nasab Zavareh et al. [23] in 2008 investigated the relationship between the severity of COPD and anemia in patients referred to Hazrat Rasool Akram Hospital. In their study, 96 patients with COPD were evaluated and concluded that the prevalence of anemia in patients with COPD is 27% and is not related to the severity of COPD. Anemia can exacerbate the limitations caused by COPD, and treating anemia can improve the quality of life of these patients.

On the other hand, Ataran et al. in 2007 investigated anemia and its relationship with serum erythropoietin levels in patients with chronic COPD. In this study, 80 patients with COPD with a mean age of 66.4 ± 11.5 years and FEV1 of 45% were included. The severity of the disease was classified based on the GOLD guidelines. The results of this study showed that anemia was observed in 13 patients (16.3%). The serum erythropoietin level was 59 ± 203 and 70.3 ± 255 units per liter in the anemic and non-anemic groups, respectively, with no significant difference between the two groups (P=0.264). Additionally, there was no relationship between anemia and the severity of COPD. Therefore, this study suggests that anemia is relatively common in patients with COPD, although the level of erythropoietin in anemic patients is elevated, but there is no significant difference between anemic and non-anemic patients [24].

On the other hand, the prevalence of hypogonadism in COPD has been reported to be between 22% and 41% in different studies [15][25][26]. It can be said that the difference in the prevalence of hypogonadism in different studies may be due to differences in racial, age, body mass index, and methods of measuring testosterone. Regardless of these differences, considering the prevalence of hypogonadism in the general population, which is reported to be around 6% [27], Zohal et al. in a study in 2015 investigated the relationship between hypogonadism and the severity of COPD. In this cross-sectional study, 57 outpatients with COPD with different severity levels were included, who had been referred to the pulmonology clinic of Qazvin province between 2012 and 2013. These patients were divided into two groups of mild and severe disease, and their serum total and free testosterone levels, FEV1, smoking status, and corticosteroid use were determined.

The data were compared using chi-square and analysis of covariance tests. The results showed that based on the level of free testosterone, 26.7% of patients in the mild group and 22.2% of patients in the severe group had hypogonadism. The mean total and free testosterone levels did not differ significantly between the two groups (3.2 ± 1.5 ng/mL and 3.9 ± 1.6 pg/mL in the mild group, and 3.1 ± 1.3 ng/mL and 4.2 ± 1.7 ng/mL in the severe group). The mean total and free testosterone levels in the two groups did not differ significantly after adjusting for age, body mass index, corticosteroid use, and smoking. They concluded that a significant proportion of patients with COPD have hypogonadism, and it seems that in clinically stable patients, serum testosterone level is not related to the severity of COPD [28]. In a study by Kaparianos et al. on 24 hospitalized patients, the level of total and free testosterone had an inverse relationship with the severity of illness (based on the Apache score) [29].

The difference in the results of the mentioned studies may be due to different patient selection criteria and sample size. The possibility of hypogonadism in the study group was directly related to the level of serum C-reactive protein. Also, in a study [15], the level of serum testosterone had an inverse relationship with C-reactive protein. Inflammatory mediators can affect the hypothalamic-pituitary axis and inhibit gonadotropins, ultimately reducing serum testosterone. However, other factors such as age, smoking, decreased arterial oxygen pressure (hypoxia), and increased arterial carbon dioxide pressure (hypercapnia) also play a role in the development of hypogonadism in COPD [30].

## Conclusion

The observed correlation between anemia and elevated testosterone levels in female COPD patients may be indicative of a broader hormonal imbalance, potentially driven by the chronic inflammation and oxidative stress characteristic of COPD. This imbalance could have significant implications for disease progression, as testosterone has been shown to play a role in regulating immune function and inflammation. Furthermore, the gender-specific nature of this relationship may be related to the differing effects of sex hormones on lung function and inflammation, highlighting the need for a more nuanced understanding of the interplay between sex hormones, inflammation, and COPD. Ultimately, a deeper understanding of this relationship could inform the development of personalized treatment strategies, tailored to the unique needs of female COPD patients, and potentially leading to improved health outcomes and quality of life. Additionally, the study's findings may also have implications for the management of anemia in COPD patients, suggesting that treatment strategies should take into account the potential hormonal consequences of anemia, and that testosterone levels should be monitored and managed accordingly.

## Conflict of Interest

None.
